# Microwave–vacuum extraction cum drying of tomato slices: Optimization and functional characterization

**DOI:** 10.1002/fsn3.3352

**Published:** 2023-04-14

**Authors:** Tayyaba Alvi, Muhammad Kashif Iqbal Khan, Abid Aslam Maan, Muhammad Rizwan, Muhammad Aamir, Farhan Saeed, Huda Ateeq, Muhammad Qasim Raza, Muhammad Afzaal, Mohd Asif Shah

**Affiliations:** ^1^ Department of Biological Systems Engineering Washington State University Pullman Washington USA; ^2^ National Institute of Food Science and Technology University of Agriculture Faisalabad Pakistan; ^3^ Department of Food Engineering University of Agriculture Faisalabad Pakistan; ^4^ Faculty of Science and Technology University of Central Punjab Lahore Pakistan; ^5^ Department of Food Science Government College University Faisalabad Faisalabad Pakistan; ^6^ Department of Economics, College of Business and Economics Kebri Dehar University Kebri Dehar Ethiopia; ^7^ Division of Research and Development Lovely Professional University Phagwara India

**Keywords:** energy consumption, mathematical models, microwave drying, waste control

## Abstract

Fruits and vegetables have shorter shelf life due to their perishable nature. Tomato, being a nutritionally rich fruit needs to be preserved for a longer period. In this context, this study was designed to dry the tomato slices through microwave–vacuum drying. This process was optimized for moisture ratio and drying rate using response surface methodology (RSM). The process was investigated at different power levels (30, 50, 80, and 100 W), pressure (0, 15, 20, and 25 inHg), and time (0, 4, 6, and 10 min) using Box–Behnken design. Results indicated that color, energy efficiency, and drying characteristics were significantly affected by changing power, vacuum levels, and processing time. Besides, nine mathematical models were applied on experimental data to deeply understand the moisture ratio of tomato slices. Amongst, Midilli model was found best to describe the drying process at 100 W and 25 inHg supported by *R* ^2^ (0.9989), RMSE (0.001), and *X* ^2^ (1.34e^−4^). This study was focused on finding the optimal combinations of power, vacuum pressure, and time for better drying and reduced wastage of the fruit owing to its perishable nature. From all the microwave powers, higher microwave power and vacuum level showed better energy consumption, energy efficiencies, color retention, and rehydration capacity.

## Graphical abstract

Vacuum drying was optimized using response surface methodology for moisture ratio and drying rate. The process was investigated at different power levels (30, 50, 80, and 100 W), pressure (0, 15, 20, and 25 inHg), and time (0, 4, 6, and 10 min) using Box–Behnken design. Results indicated that color, energy efficiency, and drying characteristics were significantly affected by changing power, vacuum levels, and processing time. This study was performed to find best combinations of power, vacuum pressure, and time for the optimum drying and reducing fruit waste generation due to its perishable nature. From all the microwave powers, higher microwave power and vacuum showed better energy consumption, energy efficiencies, better color retention, and rehydration capacity.

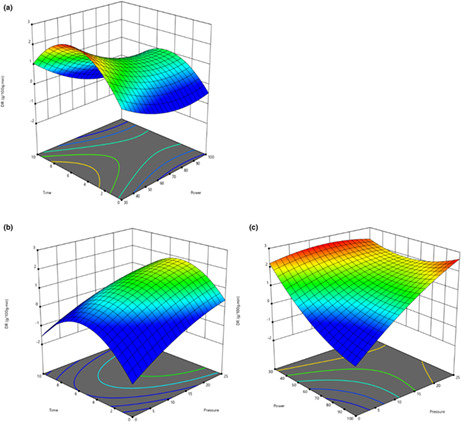

## 1 INTRODUCTION

*Solanum lycopersicum*, commonly known as tomato, is cultivated throughout the world and botanically classified as a fruit. It is an important plant matrix that has rich nutritional profile (Tan et al., 2021) like carotenoids, organic acids, proteins, vitamin E, lycopene, and various minerals (Melendres et al., 2018). These compounds possess pharmacological properties such as antioxidant activity and anti‐cancer activity and reduce chances of cardiovascular diseases (Alvi, Khan, et al., 2022; Marti et al., 2019). Tomato, being a perishable commodity (due to greater water contents), have shorter life span (2–3 days). Additionally, it is susceptible to bacterial and fungal attack, which results in a huge loss of fresh tomatoes (Sinha et al., 2019).

There is a need to preserve tomatoes for their availability throughout the year. Drying is the oldest and most convenient method for the preservation of foods which preserves the food by lowering the moisture contents that reduce microbial and enzymatic activities. During drying, weight and volume are reduced which minimizes the packaging, transportation, and storage costs. The drying affects various physicochemical properties of the product. Thus, it should be done in a such way that drying has minimal effect on the quality of product. Various drying methods are used which have their own advantages and disadvantages. Drying method is selected according to the product's physicochemical properties and economic considerations (Hafezi et al., 2016; Huang & Zhang, 2016; A. Manzoor et al., 2023). Nowadays, the attention of food processors has moved toward novel methods of drying as compared to conventional methods, e.g., sun drying, hot air drying, and tunnel drying. In this context, microwave drying, which is now considered as clean label (Alvi, Asif, et al., 2022), is extensively used to dry food materials due to high drying rate and uniform drying (Khan et al., 2021; Punathil & Basak, 2016; Sridhar et al., 2022; Zia et al., 2023). In microwave drying, the food material consisting of polar and nonpolar molecules is subjected to higher frequency electromagnetic fields, the molecules which are polar in nature rotate and move laterally millions of times per second with speeds comparable to the frequency of electromagnetic field. The interaction between adjacent molecules will interfere with and restrict the rotation of polar molecules, which thereby generates frictional heat, which accounts for major heat production in the food‐based matrices (An et al., 2022). Microwave‐based drying has the advantage that it can easily penetrate deep into the food material and reduce processing time (Ekow et al., 2013). Microwave drying obtains high drying rates along with better retention of nutrients (Ando et al., 2019). Additionally, it can also prevent shrinkage of the product (Punathil & Basak, 2016).

Besides, vacuum drying overcomes the overheating issue associated with other methods of drying by removing water at lower temperature. It avoids structure collapse and provides oxygen‐deficient environment for oxygen‐sensitive bioactive compounds (Kayisoglu & Ertekin, 2011). Therefore, microwaves combined with vacuum drying will give the benefits of both techniques; rapid heating accomplished by low‐temperature boiling environment produced by microwave and vacuum, respectively. Thus, this study was designed to optimize the combined effect of vacuum and microwave drying on drying kinetics and quality characteristics of tomato as a model product.

## 2 MATERIALS AND METHODS

### 2.1 Procurement of raw materials

Fresh and ripe tomatoes were purchased from the local market of Faisalabad, Pakistan. They were washed with running water and cut into pieces of thickness 3–5 mm with knife. After cutting, the slices were immediately subjected to microwave–vacuum drying process.

### 2.2 Microwave–vacuum drying

A novel lab‐scale setup was developed which consists of a microwave oven (OM‐30 Orient, Japan) used for microwave heating described in previous study (Khan et al., 2021). Inside, a glass chamber was used as drying reactor and connected to vacuum pump (VT‐03 Baoleide, China). The pressure of the drying chamber was manipulated by pressure regulating valve. Tomato slices (45 g by weight) were placed inside the drying cabinet and dried at various powers of microwave (30, 50, 80, and 100 W) and 0, 15, 20, and 25 vacuum levels. After drying, the samples were stored at room temperature in airtight polythene bags. A Box–Behnken design (BBD) was applied to optimize the influence of power, pressure, and time on drying of tomato slices.

### 2.3 Drying rate

The drying rate represents the drying behavior by the variation of moisture content against the drying time. The following formula was used to calculate drying rate as described by Da Silva et al. (2014).

(1) $$\text{Drying rate}=\frac{{\mathrm{MC}}_{t+dt}-{\mathrm{MC}}_{t}}{dt}$$

where ${\mathrm{MC}}_{t+dt}\;$ and ${\mathrm{MC}}_{t}$ are the values of moisture content at the time *t* + d*t* and at the time *t*, respectively.

### 2.4 Moisture ratio

Moisture contents for all the samples were converted to dimensionless moisture ratio (Rasooli Sharabiani et al., 2021). Moisture ratio (MR) was computed by the following equation;

(2) $$\text{Moisture ratio}=\frac{M}{\;M_{o}}$$

where *M* represents moisture content of the sample at any interval of time (dry basis) and $M_{O}$ represents initial moisture content of the sample on dry basis. Moreover, the experimental values of moisture ratio were compared with different thin layer models as described in the following Equations (2–10):

(3) $$\text{Midilli}\;\text{Moisture ratio}=a*\left(-kt^{n}\right)+bt$$

(4) $$\text{Page}\;\text{Moisture ratio}=\exp\left(-kt^{n}\right)$$

(5) $$\text{Henderson and Pabis}\;\text{Moisture ratio}=\exp\left(-kt\right)$$

(6) $$\text{Logarithmic}\;\text{Moisture ratio}=a*\exp\left(-kt\right)+b$$

(7) $$\mathrm{Two}-\text{Term}\;\text{Moisture ratio}=a*\exp\left(-kt+b*\exp\left(-\mathrm{nt}\right)\right)$$

(8) $$\mathrm{Two}-\text{Term Exponentials}\;\text{Moisture ratio}=a*\exp\left(-kt\right)+\left(1-a\right)*\exp\left(-k\mathrm{at}\right)$$

(9) $$\text{Wang and Sing}\;\text{Moisture ratio}=1+\mathrm{at}+bt^{2}$$

(10) $$\text{Diffusion Approximation}\;\text{Moisture ratio}=a*\exp\left(-kt\right)+\left(1-a\right)*\exp\left(-k\mathrm{bt}\right)$$

(11) $$\text{Verma}\;\mathrm{et}\;\mathrm{al}.\;\text{Moisture ratio}=a*\exp\left(-kt\right)+\left(1-a\right)*\exp\left(-\mathrm{bt}\right)$$

### 2.5 Effective moisture diffusivity (D)

Fick's second equation of diffusion was utilized to determine the effective moisture diffusivity of experimental data Equation 11. As the drying process is an unsteady state diffusion process through infinite slab, so Fick's second equation of diffusion could be written as;

(12) $$\text{Moisture ratio}=\frac{M}{M_{o}}=\frac{8}{\pi^{2}}\exp\left(\frac{-\pi^{2}\mathrm{Dt}}{4L^{2}}\right)$$

where *D* represents effective moisture diffusivity expressed in m^2^s^−1^ and *L* represents half‐thickness expressed in m, of imaginary slab of tomato slice. By plotting time versus ln MR, effective moisture diffusivity (Mahjoorian et al., 2017) can be computed by calculating the slope (*α*). The slope (*α*) can be calculated by using Equation 12:

(13) $$\alpha =\frac{-\pi^{2}D}{4L^{2}}$$

Modified Arrhenius model was utilized to estimate the dependence of *D* on microwave power levels (Chahbani et al., 2018) by using Equation (13):

(14) $$D=D_{f}\exp\left(-\frac{E_{a}m}{P}\right)$$

where *D*_*f*_ is the pre‐exponential factor of the Arrhenius equation expressed in (m^2^s^−1^), *E*_*a*_ is the activation energy expressed in Wkg^−1^, m is the average of sample mass expressed in kg, and *P* represents microwave power output expressed in *W*. Additionally, the Equation (13) was demonstrated in a logarithmic form and ln(*D*) was plotted as a function of (*m*/*P*). The slope of the plot signifies the values of *E*_*a*_ and *D*_*f*_ (Chahbani et al., 2018).

### 2.6 Specific energy consumption (SEC)

SEC is the energy used to evaporate 1 kg of water from the sample and calculated by the method of Alvi et al. (2019) and Darvishi et al. (2013) by Equation (15).

(15) $$\mathrm{SEC}=\frac{3.6\times E_{m}}{\left(M_{o}-M_{t}\right)m_{s}}$$

The microwave energy (*E*_*m*_) required to dry the tomato slices was determined by applying the equation:

(16) $$E_{m}=p\times t$$

where *m*_*s*_ is the dry matter mass in kg, *p* is microwave power in watts, and *t* is the time in seconds.

### 2.7 Energy efficiency

Energy efficiency was defined as the ratio of energy utilized to evaporate water from the surface of sample to the total energy consumed,

(17) $$\text{Energy efficiency}=\frac{Q_{w}}{{\mathrm{EU}}_{\text{ther}}+{\mathrm{EU}}_{\mathrm{mec}}}$$

$Q_{w}$ is the energy used for moisture evaporation and is given as

(18) $$Q_{w}=h_{\mathrm{fg}}.M_{w}\;$$

where $h_{\mathrm{fg}}\;\text{is}$ the heat of vaporization of water at a given temperature and $M_{w}$ is the weight of moisture loss (Harchegani et al., 2016).

### 2.8 Rehydration capacity

Dried sample of about 5 g was immersed into distilled water in the water bath at 25°C. The sample was weighed every 30 min until constant weight was obtained. After that, sample was removed and sieved through a perforated mesh to escape attached water particles and weighed after 20 min. Rehydration capacity was calculated by using the following equation as described by Borquez et al. (2015).

(19) $$\text{Rehydration capacity}=\frac{M_{\mathrm{rh}}}{M_{d}}$$

where $M_{\mathrm{rh}}$ and $M_{d}$ are the mass of rehydrated sample and dried sample, respectively.

### 2.9 Color

The surface color of the sample was measured by colorimeter (COLOR TECH PCM). Color values were recorded by Hunter lab chromatic system and measured in terms of L* (whiteness or darkness), a* (greenness or redness), and b* (yellowness or blueness). Change in color after drying was referred to as total change in color (Δ*E*) and was calculated by the equation as measured by Alvi et al. (2019) and Manzoor et al. (2019). However, chroma and whiteness index were calculated by Equations 17 and 18.

(20) $$\text{Change in color}\;\left(\Delta E\right)=\sqrt{{\left(∆L^{*}\right)}^{2}+{\left(∆a^{*}\right)}^{2}+{\left(∆b^{*}\right)}^{2}}$$

(21) $$\text{Chroma}\;\left(C^{*}\right)=\sqrt{\;{\left(\;a^{*}\;\right)}^{2}+{\left(b^{*}\right)}^{2}}\;$$

(22) $$\text{Whitness index}\;\left(\mathrm{WI}\right)=100-\sqrt{{\left(100-L^{*}\right)}^{2}+a^{*2}+b^{*2}}$$

### 2.10 Statistical analysis

In this study, Box–Behnken design (BBD) was chosen for the modeling of processing variables (power, time, and pressure). The regression models were constructed for three parameters and coded levels of these parameters along with experimental design are presented in Table 1. Moreover, second‐order polynomial equation was fitted for drying rate and moisture ratio. Response surface methodology (RSM) was applied to evaluate the influence of power and pressure on drying rate and moisture ratio of tomato slices.

**TABLE 1:** Actual and coded levels of independent variables for optimization of vacuum‐assisted microwave drying of tomato slices as determined by Box–Behnken design.

Variables	(un) coded levels			
	−1	−0.33	0.33	1
Power	30	50	80	100
Pressure	0	15	20	25
Time	0	4	6	10

Experiments were carried out in triplicate and data of parameters were shown as corresponding mean values along with standard deviation. The quadratic equation was utilized to explain the behavior of Box–Behnken design. Design Expert (Stat‐Ease, Inc) was used to determine the level of significance of drying rate and moisture ratio. A 5% level of significance was used to analyze significant differences in treatments (Nurmitasari & Mahfud, 2021). Statistical tests were carried out that are described in the following equations:

(23) $$\text{Root mean square error}\;\left(\text{RMSE}\right)=\sqrt{\frac{1}{N}\sum_{i=1}^{n}{\left(V_{\exp,i}-V_{\text{model},i}\right)}^{2}}$$

(24) $$\mathrm{Sum}\;\text{of square error}\;\left(\mathrm{SSE}\right)=\frac{1}{N}\sum_{i=1}^{n}{\left(V_{\exp,i}-V_{\text{model},i}\right)}^{2}$$

(25) $$\mathrm{Chi}\;\text{square}\;\left(\mathrm{CS}\right)=\frac{1}{N-n_{p}}\sum_{i=1}^{n}{\left(V_{\exp,i}-V_{\text{model},i}\right)}^{2}$$

(26) $$\text{Relative percent deviation}\;\left(\mathrm{RPD}\right)=\frac{100}{N}\sum_{i=1}^{n}\frac{\left|V_{\exp,i}-V_{\text{model},i}\right|}{V_{\exp,i}}$$

where *N* represents the number of observations, *n*_*p*_ indicates the number of parameters, *V*_exp,*i*_ and *V*_model,*i*_ are the experimental and model values of *i* ^th^ observation, respectively. Best model was selected based on the coefficient of determination (*R* ^2^). Moreover, root mean square error, Chi‐square (CS), and relative percent deviation (RPD) indicate variation (goodness of fit) in model and experimental values. RMSE and CS values closer to zero indicate the closeness of model to experimental data. Similarly, RPD determines the absolute difference between model and experimental values. RPD value <10% indicates that fit is good (Mota et al., 2010; Roberts et al., 2008).

## 3 RESULTS AND DISCUSSIONS

### 3.1 Microwave–vacuum‐assisted drying of tomato slices

Different factors such as power levels (W), pressure (Psi), and time (sec) were studied for the optimization of vacuum–microwave‐assisted drying process for tomato slices by using four‐level three‐factor Box–Behnken design. According to this design, totally 20 runs were carried out and the values of drying rate and moisture ratio obtained by these runs are shown in Table 2. The highest drying rate value (2.59 g/100 g·min) was observed at 80 W and 25 inHg for 4 min of processing time, while lowest value was (0.07 g/100 g·min) at 80 W power and 25 inHg pressure for 10 min of processing time.

**TABLE 2:** Drying rate and moisture ratio as carried out by the Box–Behnken design.

Runs	Variables			DR (g/100 g·min)	MR
	Power (W)	Pressure (psi)	Time (min)		
1	30	0	0	0	1
2	50	25	6	1.27	0.583
3	30	25	10	0.50	0.526
4	50	15	0	0	1
5	50	25	0	0	1
6	80	25	4	2.59	0.357
7	30	25	10	0.50	0.526
8	30	25	4	2.50	0.864
9	100	20	6	1.96	0.171
10	80	25	4	2.59	0.357
11	80	25	10	0.07	0.118
12	80	25	10	0.09	0.118
13	100	15	0	0	1
14	30	25	4	2.54	0.864
15	100	25	6	2.20	0.171
16	50	25	6	1.27	0.583
17	100	15	0	0	1
18	30	25	4	2.54	0.864
19	100	25	6	2.25	0.171
20	50	25	10	0.50	0.401

### 3.2 Drying rate

A model equation, obtained through RSM analysis, in terms of processing variables is expressed in Equation (1) and ANOVA results are presented in Table 3. The *p* value (.0002) indicated that model equation was highly significant at *p* ≤ .05. Quadratic model had coefficient of variation of 36.93%. However, coefficient of determination (*R* ^2^) of 0.919 and the adjusted *R* ^2^ of 0.846 were close as the difference was ~0.073 (Chakraborty et al., 2011). The adjusted *R* ^2^ is the variance proportion in the output that is predictable from the input that in the real sense had effects on the output, whereas predicted *R* ^2^ is an indicator of how well a regression model predicts outputs for new observations. Thus, this statistical evaluation indicates that the model equation can be used to navigate space.

**TABLE 3:** ANOVA table for drying rate.

	Source	Sum of squares	DF	Mean square	*F*‐value	*p*‐value	
	Model	21.130	9	2.3500	12.61	.0002	Significant
Linear	A‐Power	0.2144	1	0.2144	1.15	.3085	
	B‐Pressure	0.0927	1	0.0927	0.498	.4965	
	C‐Time	0	1	0	0.0002	.9879	
Interaction	AB	0.2242	1	0.2242	1.2	.2982	
	AC	0.0807	1	0.0807	0.4331	.5253	
	BC	0.0045	1	0.0045	0.0244	.8789	
Quadratic	A^2^	0.6355	1	0.6355	3.41	.0945	
	B^2^	0.0288	1	0.0288	0.1549	.7021	
	C^2^	5.86	1	5.86	31.48	.0002	Significant
	Residual	1.86	10	0.1862			
	Lack of fit	1.86	2	0.9298	2955.64	<.0001	Significant
	Pure error	0.0025	8	0.0003			
	Cor total	22.99	19				

Drying rate (DR) value was maximum reached to 11 g/100 g·min in 6 min at 100 W compared to 30 W (4 g/100 g·min). With the decrease in power from 100 to 30 W, the DR value was reduced up to three times which increase the overall processing time. This signifies that drying rate increases with increase in microwave power level and reduces with decrease in microwave power level and vice versa. Additionally, the time required to reach the maximum values of drying rate at different power levels (100, 80, 50, and 30 W) varied significantly. This behavior can be attributed to rapid excitation of physically bounded water molecules which leads to rapid evaporation of these molecules.

Moreover, drying rate initially increased to its maximum and then started to decrease with further processing. This implies that drying rate observed parabolic behavior during the whole process irrespective of power levels. A falling rate period was observed that was caused by the initial rapid removal of moisture from the tomato slices. However, the rate of drying slowed with increase in drying time. This behavior (reduction in rate) may be due to less amount of water present in the tomato slices in the second period of drying. Many researchers reported reduction in drying rate due to a decrease in moisture contents (Alvi et al., 2019; Khan et al., 2016; Mahjoorian et al., 2017).

$$\mathrm{Dr}=2.547–0.098846\;P+0.01381\;\mathrm{Pr}+0.823887\;T+0.001945\;P^{*}\mathrm{Pr}–0.001269\;P^{*}T–0.003267\;{\mathrm{Pr}}^{*}T+0.000465\;P^{2}–0.00192\;{\mathrm{Pr}}^{2}–0.070423\;T^{2}.$$

where P is the power, Pr is the pressure, and T is the time.

**FIGURE 1:**
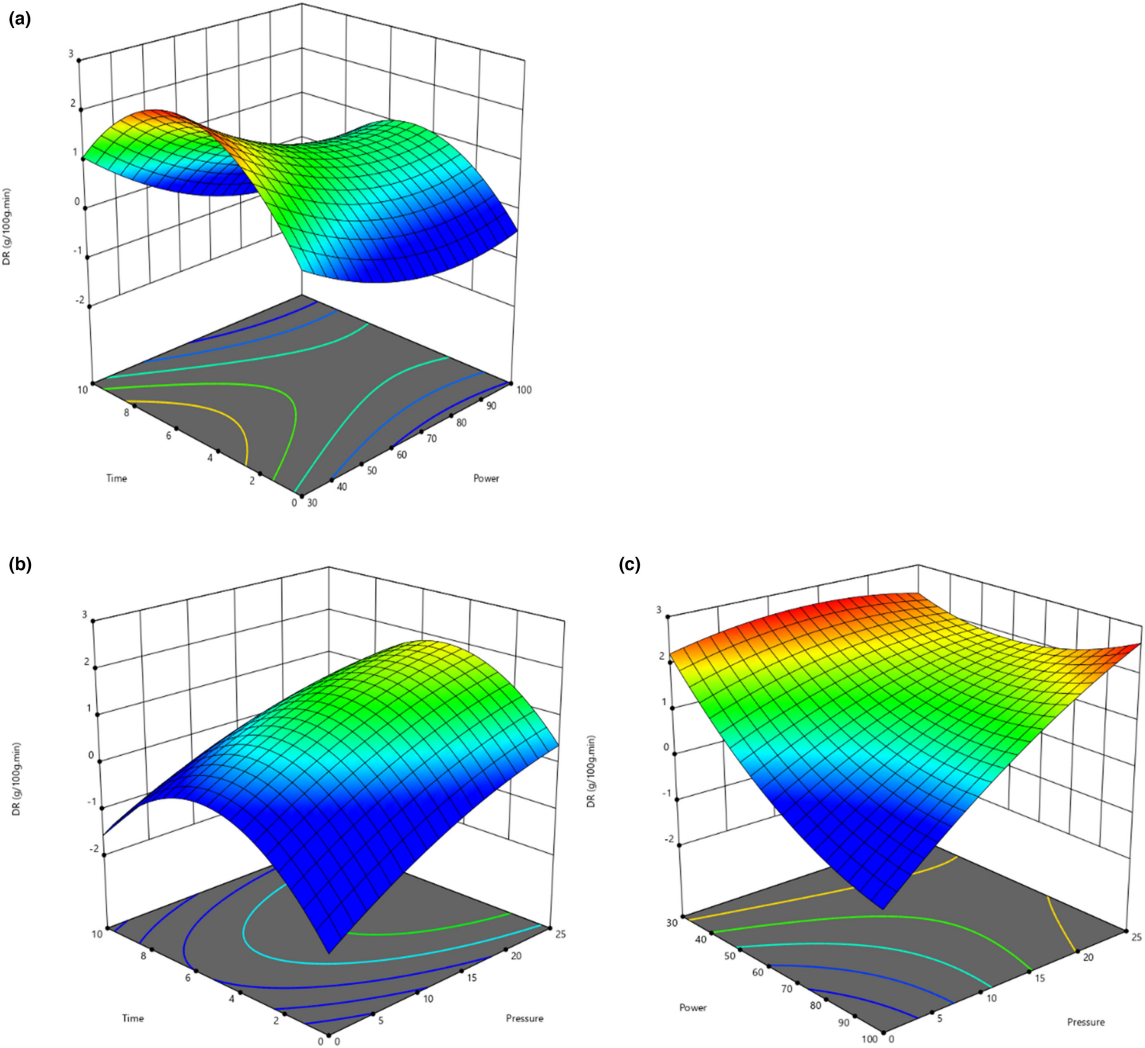
Drying rate of tomato slice as a function of power, time, and pressure (a–c) during MADE processing.

Likewise, drying rate was significantly increased with increase in vacuum pressure in the drying chamber (Figure 1b). The experimental results of combined effect of power and vacuum showed that at highest level of vacuum and pressure (25 inHg and 100 W), the drying rate elevated to 11.38 g/100 g·min as compared to lowest level of vacuum and pressure (15 inHg and 30 W) which observed to be 2.55 g/100 g·min (Figure 1c). This might be due to continuation of moisture gradient inside the drying chamber by application of vacuum. Later one facilitates the rapid extraction of the moisture produced during drying process. Thus, drying rate exhibited a linear relationship with the increase in vacuum inside the drying chamber. These results are supported by the literature (Ambros et al., 2018) which proves increase in vacuum pressure there is distinct increase in drying rate (Xie et al., 2018).

### 3.3 Moisture ratio

A model equation, obtained through RSM analysis, in terms of processing variables is expressed in Equation (2) and ANOVA results are presented in Table 4. The *p* value (<.0001) indicated that model equation was highly significant at *p* ≤ 0.05. Quadratic model had coefficient of variation (COV) of 5.62%. However, coefficient of determination (*R* ^2^) of 0.995 and the adjusted *R* ^2^ of 0.991 were close as the difference was ~0.004 (Chakraborty et al., 2011). The adjusted *R* ^2^ is the variance proportion in the output that is predictable from the input that in the real sense had effects on the output, whereas predicted *R* ^2^ is an indicator of how well a regression model predicts outputs for new observations. Thus, this statistical evaluation indicates that the model equation can be used to navigate space.

**TABLE 4:** ANOVA table for moisture ratio.

	Source	Sum of squares	Df	Mean square	*F*‐value	*p*‐value	
	Model	2.18	9	0.2421	225.13	<.0001	Significant
Linear	A‐Power	0.0057	1	0.0057	5.31	.0439	Significant
	B‐Pressure	0.0015	1	0.0015	1.35	.2725	
	C‐Time	0.0713	1	0.0713	66.3	<.0001	Significant
Interaction	AB	0.0562	1	0.0562	52.29	<.0001	Significant
	AC	0.0036	1	0.0036	3.37	.0962	
	BC	0.0215	1	0.0215	20.02	.0012	Significant
Quadratic	A^2^	0.0014	1	0.0014	1.30	.2814	
	B^2^	0.0028	1	0.0028	2.63	.1359	
	C^2^	0.0071	1	0.0071	6.58	.0281	Significant
	Residual	0.0108	10	0.0011			
	Lack of fit	0.0108	2	0.0054			
	Pure error	0	8	0			
	Cor total	2.19	19				

**FIGURE 2:**
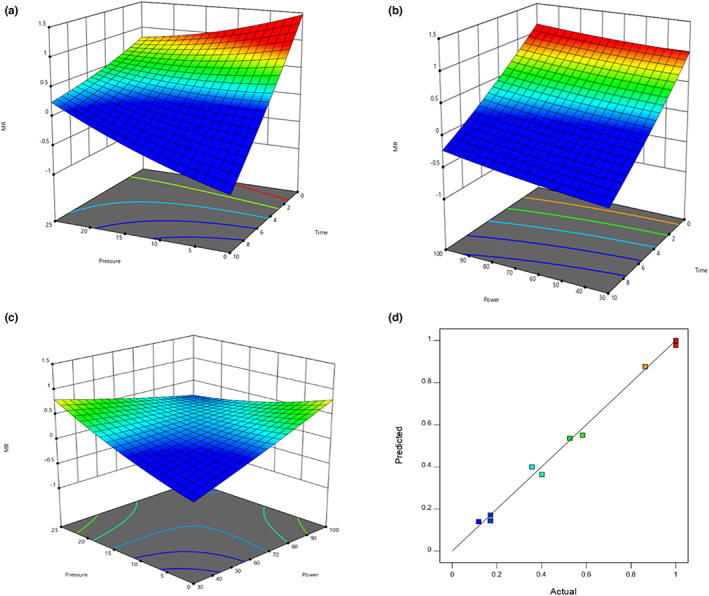
(a–c) Moisture ratio as a function of power, pressure, and time (a–c). While Figure (d) represents the comparison between predicted (line) and actual moisture ratio (square).

Moisture ratio depicts the relative moisture loss from the sample and helps to elaborate the drying kinetics. Figure 2 shows the changes of the moisture ratio for tomato slices dried by vacuum microwave drying at various microwave power levels (30, 50, 80, and 100 W) and 0, 15,20, and 25 inHg pressure levels. At 25 inHg of vacuum, the moisture ratio maximum reduced to 0.08 at 100 W power in 5 min; however at 30 W, there observed a 0.52 moisture ratio after same time interval (5 min).

Besides, vacuum pressure ease the drying process due to which there observed the least values of MR at higher vacuum pressures (25 inHg), while keeping power constant. For example, at 50 W power, moisture ratio observed to be 0.6, 0.5, and 0.4 for 25, 20, and 15 inHg, respectively. Increasing the microwave power and vacuum level rapidly decreases the moisture ratio of slices (Khan et al., 2016). High power and pressure levels might evaporate the moisture more rapidly; subsequently reducing the moisture ratio values. The experimental findings are in line with Ali et al. (2020) who studied the drying kinetics of *Rosmarinus officinalis* L. through vacuum microwave drying.

The analysis of variance showed that the effect of power and time was significant on MR values (Table 4). Moreover, the combined study of these three variables exhibited that in combined effect of power, pressure, and time, pressure has significant impact (Figure 2). The moisture ratio values were predicted through various models (Equations 3–11). The experimental and predicted moisture ratio were compared and both values were good fit with each other (Figure 2d).

At all microwave powers, it was clear that the moisture ratio decreases with the passage of time, but at variable rates. However, a significant difference in the moisture ratio curves plotted at different microwave powers was observed. This effect can be due to swift/rapid heating at elevated power levels. Therefore, there is a rapid decrease at higher power levels in moisture ratio of tomato slices, which leads to steeper curve in comparison with drying at lower power levels. As the moisture ratio decreases rapidly at higher microwave powers and drying rate also maximum at higher microwave powers, it was suitable to study the mathematical modeling at 100 W power.

The experimental moisture ratio was compared by applying nine thin layer models. The model which had value of *R* ^2^ close to 1 and RMSE lower than 0.01 demonstrated appropriateness to describe the drying kinetics of tomato slices. Statistical analysis revealed that from all the models applied Midilli model was found to be the most appropriate model to describe the drying characteristics of tomato slices, with highest *R* ^2^ (0.998) and lowest RMSE (0.001), *χ* ^2^ (1.34 × 10^−4^) values for drying at 100 microwave power (Table 5). Moreover, the experimental moisture ratio values were compared with Midilli model values (Figure 2c) and found similar trend in both values. From drying kinetics, it was concluded that tomato slices exhibited better drying behavior during microwave processing at 25 inHg psi vacuum. Thus, further experiments were performed at this optimized pressure (25 inHg).

$$\mathrm{MR}=0.640058+0.011345\;P+0.022229\;\mathrm{Pr}–0.276805\;T–0.000974\;P^{*}\mathrm{Pr}+0.000269\;P^{*}T+0.007107\;{\mathrm{Pr}}^{*}\;T+0.000022\;P^{2}+0.000601\;P^{2}+0.002448\;T^{2}.$$

where P is the power, Pr is the pressure, and T is the time.

**TABLE 5:** Statistical determinations of thin layer models for microwave‐assisted drying of tomato slices at 100 W.

Model name	Model constants				*R* ^2^	CHI (*χ* ^2^)	SSE	RMSE	RPD
	*K*	*a*	*b*	*n*					
Henderson and Pabis	0.277	1.06	–	–	0.9879	1.0e^−3^	0.013	0.036	3.716
Diffusion approximation	0.16	76.322	0.994	–	0.9868	2.0e^−3^	0.014	0.038	2.764
Logarithmic	0.254	1.089	−0.039	–	0.9889	1.0e^−3^	0.012	0.035	1.349
**Midilli**	**0.172**	**1.008**	**0.007**	**1.392**	**0.9989**	**1.34e** ^**−4**^	**0.001**	**0.001**	**0.111**
Page	0.187	–	–	1.235	0.9944	6.74e^−4^	0.006	0.025	7.881
Two‐term exponential	0.381	1.845	–	–	0.9953	5.71e^−4^	0.005	0.023	9.005
Two‐term	0.277	0.488	0.572	0.277	0.9879	1.0e^−3^	0.013	0.036	3.716
Verma et al.	0.16	80.91	0.159	–	0.9868	2.0e^−3^	0.014	0.038	2.764
Wang and Sing	–	−0.207	0.012	–	0.994	7.26E^−4^	0.007	0.026	0.749

*Note*: Bold values only represents that the result of this model is suitable for this study and it explains the best drying behaviour. The higher vaue of R and lower value of RMSE describes that this model suits best to the present study.

### 3.4 Effective moisture diffusivity

The effective moisture diffusivities (*D*) were computed by using graphical method. Slope of the straight line led to the determination of *D* at all microwave powers. Effective moisture diffusivity ranges from 0.852 to 3.32 × 10^−14^ (m^2^/s) with the change in microwave power from 30 to 100 W (Table 6). When power level increases from 30 to 50, the value of effective diffusivity increases but there was not a significant increase observed. Furthermore, when power increases from 50 to 80 W, a 68% increase in effective diffusivity was observed. Further increase in power (from 80 to 100 W) caused an 18% increase in diffusivity. Thus, this can be concluded that effective diffusivity increased with increase in microwave power levels. This increase in values of D may be due to a rapid increase in product temperature and consequently a rise in the water vapor pressure. Similar changes in *D* values were reported elsewhere in the literature (Alvi et al., 2019; Zarein et al., 2015).

**TABLE 6:** Effect of power on activation energy, effective diffusivity, specific energy consumption, and energy efficiency at constant pressure.

Power (W)	Activation energy (×10^−4^ w/kg)	Effective diffusivity (×10^−14^ m^2^/s)	Specific energy consumption (MJ/kg)	Energy efficiency (%)
30	3.046 ± 0.02	0.852 ± 0.01	0.237 ± 0.05	14.09 ± 0.04
50	1.813 ± 0.06	1.854 ± 0.01	0.314 ± 0.07	17.40 ± 0.05
80	1.928 ± 0.05	2.720 ± 0.03	1.681 ± 0.02	23.50 ± 0.09
100	1.640 ± 0.05	3.320 ± 0.04	3.135 ± 0.08	26.55 ± 0.02

Additionally, the activation energy (*E*_*a*_) was computed from modified Arrhenius plot of ln (*D*) as a function of ratio of sample mass and microwave power (m/p) (Equation (14)). The value of activation energy was 3.04 e^−4^ w/kg at lowest microwave power (30 W). While a 40% reduction in activation energy was observed when power was changed from 30 to 50 W. A further increase in power level had further slashed the value of *E*_*a*_. This decrease was nonsignificant compared to 50 and 80 W. Thus, it was obvious that activation energy decreased as the power increases. It can be observed from Table 6 that activation energy and effective diffusivity are reciprocal to each other, as activation energy decreases, the power increases and effective diffusion increases as the power increases. So, this can be concluded that during microwave drying, higher power has more activation energy to evaporate the water molecules more quickly (Chahbani et al., 2018).

### 3.5 Specific energy consumption

Specific energy consumption was calculated by an Equation (15) and results are demonstrated in Table 6. Specific energy consumption depends upon the power absorbed by the sample, drying time, and vacuum applied (Jindarat et al., 2013). The specific energy consumption is varied from 0.2367 to 3.1349 MJ/kg in the experimental power levels. The highest energy consumption was observed for 100 W as 3.1349 MJ/kg and lowest for 30 W. The SEC value increased 15 times when power was increased from 30 W to 100 W, respectively. However, when power increased from 50 to 100 W the specific energy consumption increased 10 times. But when power increased from 80 to 100 W, the specific energy consumption increased two times. The reason for higher energy consumption at 100 power was the time period to which samples were subjected for drying remained the same while power level was higher. The experimental results showed similar trend as reported previously that there is higher energy consumption for lower microwave powers and lower energy consumption for higher microwave powers (Stepien et al., 2019).

### 3.6 Energy efficiency

The energy efficiency of vacuum microwave drying of tomato slices was calculated (Equations 17, 18) and results are presented in Table 6. It is evident that 100 W has higher energy efficiency (26.5%) followed by 80 W. As the microwave power decreases, the energy efficiency tends to lower in values and vice versa. This shows efficiency decreased significantly (46%) when microwave power decreased from 100 to 30 W. This effect may be due to higher process time for the drying of tomato slices. Torki and his coworkers studied the dehydration behavior of peppermint leaves and found similar results (Harchegani et al., 2016). Zarein et al. (2015) also studied the energy efficiency of apple slices and observed similar behavior regarding energy efficiency.

### 3.7 Rehydration capacity

**FIGURE 3:**
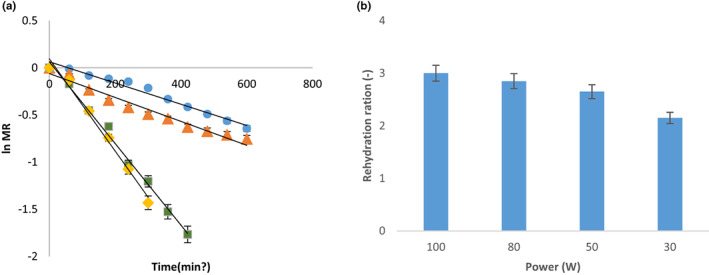
Natural log of moisture ratio as a function of time (a) and rehydration ratio of tomato slices as a function of power (b) at constant pressure.

Rehydration capacity is the ability of dried sample to absorb water. In this study, maximum rehydration ratios were found to be 3 for 100 (W) microwave power and minimum was observed for 30 W (Figure 3b). The tomato slices dried at 100 W power show highest rehydration capacity of 3 and remaining trend is 2.85, 2.65, and 2.15 observed for 80, 50, and 30 W, respectively. This signifies that high microwave powers have higher rehydration capacities and lower microwave powers have lower rehydration capacities. This can be due to reduced structural collapse during drying at higher microwave powers, which can be due to heating time reduced to greater extent at higher microwave powers. High value of rehydration capacity represents better quality of dried sample because higher rehydration means less tissue and structural damage (Izli & Polat, 2019) while low value of rehydration capacity showed that more tissues were damaged by drying process because water cannot be absorbed, as pores of tissues are closed due to tissue damage. Thus, study results indicated that higher power posed less damage to cell structure. The results are comparable to the findings of Tepe and Tepe (2020).

### 3.8 Color

Color changes of tomato slices after microwave treatment were determined as shown in Table 7. The color difference of the samples with and without microwave application was in the range of 2.94–5.20. The samples dried at higher microwave power such as 100 W have comparatively lower change in color. However, the lower powers have greater change in color (Table 7). The chroma describes the strength of a color. Higher value of chroma describes that there is lesser presence of gray color. The chroma values are significantly decreased as the microwave power decreased. The chroma values increased one time when power increased from 30 to 100 W. However, higher values of chroma were observed for higher microwave powers. This describes that grayness is reduced as the power increases. Whiteness index (WI) of microwave powers was comparatively higher as compared to control samples. Moreover, whiteness index of samples dried at 100 W is closer to control samples. But, as the power decrease to 30 W from 100 W, whiteness index increases one time. Similar trend was observed by Orikasa et al. (2018) when vacuum microwave drying was observed for tomato fruit.

**TABLE 7:** Color analysis for tomato slices dried through hot air oven and microwave at 100 W and constant pressure.

Power	ΔE	Chroma	WI
Control	0	11.30 ± 0.19	44.88 ± 0.03
100	2.94 ± 0.05	9.34 ± 0.05	47.25 ± 0.05
80	4.19 ± 0.02	8.52 ± 0.01	48.45 ± 0.01
50	5.15 ± 0.06	7.88 ± 0.06	49.30 ± 0.02
30	5.20 ± 0.15	7.80 ± 0.82	49.41 ± 0.01

## 4 CONCLUSION

The effect of vacuum microwave drying on drying kinetics, rehydration capacity, and color of tomato slices was investigated in this study. Vacuum microwave drying reduced the total processing time for drying of tomato slices. However, vacuum had a significant effect at 25 inHg pressure, which can be due to the achievement of higher temperature for drying at extremely reduced time. Response surface methodology was applied, in which microwave power levels and vacuum pressure were optimized through quadratic models by using Box–Behnken design. The results from response surface methodology indicated that higher microwave powers and higher vacuum levels showed better drying characteristics as compared to lower microwave powers. The experimental data of MR were subjected to nine empirical mathematical models, from which Midilli model (*R* = 0.9989) best described the drying of tomato slices. Moreover, tomato slices dried at 100 W showed better color acceptability indicated by Δ*E*, WI, and C*. Additionally, drying at 100 W gives the most appropriate rehydration capacities. The results of the current research can be important by providing information to study drying characteristics and behavior during microwave drying process of tomato slices and other similar fruits and vegetables.

## AUTHOR CONTRIBUTIONS

**Tayyaba Alvi:** Conceptualization (supporting); formal analysis (lead); writing – original draft (lead). **Muhammad Kashif Iqbal Khan:** Conceptualization (lead); supervision (lead); writing – original draft (equal). **Abid Aslam Maan:** Methodology (equal); supervision (equal); visualization (equal). **Muhammad Rizwan:** Investigation (equal); methodology (equal). **Muhammad Aamir:** Investigation (equal); methodology (equal). **Farhan Saeed:** Data curation (equal); writing – review and editing (equal). **Huda Ateeq:** Software (equal). **Qasim Raza:** Software (equal). **Muhammad Afzaal:** Formal analysis (equal); visualization (equal). **Mohd Asif Shah:** Writing – review and editing (equal).

## FUNDING INFORMATION

The authors declare that no funds, grants, or other support were received during the preparation of this manuscript.

## CONFLICT OF INTEREST STATEMENT

The authors declare that they have no conflict of interest.

## ETHICS STATEMENT

This article does not contain any studies with human participants or animals performed by any of the authors.

## CONSENT TO PARTICIPATE

Corresponding and all the co‐authors are willing to participate in this manuscript.

## CONSENT FOR PUBLICATION

All authors are willing for publication of this manuscript.

## Data Availability

Even though adequate data have been given in the form of tables and figures; however, all authors declare that if more data required then the data will be provided on request basis.
